# Study on the chemical stability of β-lactam antibiotics in concomitant simple suspensions with magnesium oxide

**DOI:** 10.1186/s40780-024-00396-0

**Published:** 2024-11-18

**Authors:** Ginjiro Kato, Hidemichi Mitome, Syu Takeda, Noriaki Hidaka, Mamoru Tanaka, Kazuki Akira

**Affiliations:** 1 Laboratory of Pharmaceutical Analytical Chemistry, College of Pharmaceutical Sciences, Matsuyama University, 4-2 Bunkyo-cho, Matsuyama, Ehime 790-8578 Japan; 2 Division of Pharmacy, Ehime University Hospital, 454 Shitsukawa, Toon, Ehime 791-0295 Japan

**Keywords:** Simple suspension method, Magnesium oxide, Dysphagia, Co-suspension, β-lactam antibiotics, Degradation

## Abstract

**Background:**

A simple suspension method, where solid formulations are disintegrated and suspended by being soaked in warm water followed by tube administration, is widely used, especially for elderly patients with dysphagia in Japanese clinical settings. However, there is insufficient information on drug stability in the simple co-suspension of multiple formulations especially including acidic or alkaline ones. The influence of occasional prolonged soakage on drug stability is also of concern. In this study, the chemical stability of typical β-lactam antibiotics, amoxicillin, and cefcapene pivoxil hydrochloride, was investigated in simple co-suspensions with magnesium oxide (MgO), which is frequently used as an alkaline laxative for the elderly.

**Methods:**

Amoxicillin (capsule) or cefcapene pivoxil hydrochloride (tablet) was placed with or without MgO (tablet) in a centrifuge tube containing warm water (55 °C). The tube was allowed to stand for 10 min or 5 h at room temperature and simple suspensions were prepared. The suspensions were then treated with large amounts of solvents and neutralized using a weakly acidic cation exchange resin. The resulting solutions were analyzed by high-performance liquid chromatography. The degradation products were identified by mass spectrometry and nuclear magnetic resonance spectroscopy.

**Results:**

Amoxicillin was found to be partially degraded to amoxicilloic acid and amoxicillin diketopiperazine by the co-suspension with MgO. The degree of degradation increased with the prolonged soaking. The recovery rates of cefcapene pivoxil decreased due to the poor solubility in the co-suspensions with MgO and no degradation product of the drug was observed.

**Conclusions:**

Amoxicillin and MgO should be independently suspended because of the chemical instability of amoxicillin. This study has also indicated there is a degradation risk after prolonged soaking. It should be noted that the poor water solubility of cefcapene pivoxil under alkaline conditions may affect the absorption process as well as tube passability.

**Supplementary Information:**

The online version contains supplementary material available at 10.1186/s40780-024-00396-0.

## Introduction

Dysphagia, a swallowing dysfunction, is a common issue for the elderly population since such medical conditions make it difficult to take solid oral formulations as well as food [1, 2]. A simple suspension method (SSM) has thus been developed and widely used in Japanese clinical settings in order to administer solid formulations to patients with dysphagia, particularly for elderly ones. In the SSM, solid formulations are soaked in warm water, by which they are spontaneously disintegrated and easily suspended for tube administration. Thus, the SSM does not require pulverization of tablets nor opening of capsules, and loss of drugs and drug exposure to clinical staff can be avoided [3–5]. Although the SSM has been reported to be applicable for most solid formulations (90%) [6], there are concerns about the chemical stability of drugs under the condition. In particular, there is insufficient information on chemical compatibility upon mixing of multiple formulations including acidic or alkaline drugs. Such information is especially important for elderly patients who take multiple drugs at the same time. Furthermore, the influence of occasional prolonged soaking on drug stability is also of concern.

Magnesium oxide (MgO) has been widely used as a laxative in East Asia [7, 8]. Safety information about careful administration of MgO to the elderly has been previously shared by the Pharmaceuticals and Medical Devices Agency (of Japan) because of the risk of hypermagnesemia [9]. However, over time, old age was reported to be not a significant risk factor although careful administration is required for patients with renal dysfunction [10, 11]. In this context, the use of the drug has become frequent due to an increase in the number of elderly patients with chronic constipation in Japan, a super aging society [12]. MgO is slightly soluble in water and shows basicity and Mg^2+^ generated from MgO has the ability to form complexes or chelates [13–15]. Thus, MgO possibly affects the chemical stability of other drugs in concomitant simple suspensions (hereafter simply called ‘co-suspension’). In fact, several drugs have been reported to degrade in co-suspensions with MgO [16–18].

Antibiotics are frequently prescribed to elderly patients, as they are commonly susceptible to infectious diseases due to a decline in immunologic function [19]. β-lactam antibiotics are one of the most widely used antibiotic classes [20]. The chemical stability of β-lactam antibiotics under SSM conditions with MgO is questionable, because the cyclic amide ring condensed with an additional one has a higher tension [21, 22]. Amoxicillin and cefcapene pivoxil hydrochloride are typical penicillins and cephems, respectively [23]. Thus, in this paper, the chemical stability of these β-lactam antibiotics was investigated in co-suspensions with MgO.

## Methods

### Reagents

Amoxicillin capsules 250 mg (Towa, Osaka, Japan), Cefcapene pivoxil hydrochloride tablets 100 mg (Sawai, Osaka, Japan), and Magmitt tablets 330 mg (Nihon Shinyaku, Kyoto, Japan) as an MgO containing formulation were used. These formulations of amoxicillin, cefcapene pivoxil hydrochloride, and MgO are hereafter referred to as AMX, CFPN, and MG, respectively. Amoxicillin trihydrate (100%) and cefcapene pivoxil hydrochloride monohydrate (98.5%) were purchased from Tokyo Chemical Industry (Tokyo, Japan). Distilled water, acetonitrile, methanol, and trifluoroacetic acid (TFA) were purchased from Fujifilm Wako Chemicals (Osaka, Japan). These solvents were of high-performance liquid chromatography (HPLC) grade. Weakly acidic cation exchange resin AMBERLITE^®^ (IRC76) was purchased from ORGANO (Tokyo, Japan). Membrane filters, hydrophobic DISMIC^®^ − 03 JP (0.50 μm) and hydrophilic DISMIC^®^ − 03 CP (0.45 μm), were purchased from Toyo Roshi Kaisha (Tokyo, Japan). Deuterated dimethyl sulfoxide (DMSO-*d*_6_) was purchased from Kanto Chemical (Tokyo, Japan).

### Preparation of simple suspensions

The simple suspensions were basically prepared according to the previously described method [6, 17]. AMX (one capsule) or CFPN (one tablet) was soaked with or without MG (one tablet) in a 50-mL plastic centrifuge tube containing 20 mL of warm distilled water (55 °C). The centrifuge tube was allowed to stand at room temperature (ca. 25 °C) for 10 min or 5 h. The centrifuge tube was wrapped with aluminum foil for shading during standing. The mixture was then mixed with inversion 15 times and vortexed for one min to prepare a suspension, which was treated and analyzed as shown below. In separate experiments, the co-suspension of AMX and MG was prepared after 10 min soakage, and the aqueous supernatant after centrifugation was directly analyzed by HPLC and subjected to pH measurements using InLab^®^ Routine Pro (Mettler Toledo, Ohio, USA). The suspension preparation was performed three times for each antibiotic in all of the experiments.

### Treatment of simple suspensions

The suspension of AMX with or without MG in a centrifuge tube was completely transferred into a beaker using 180 mL of distilled water. After sonication for 2 min, the suspension was centrifuged (13000 × *g*) for 5 min. The aqueous supernatant obtained was passed through a column packed with weakly acidic cation exchange resin (ca. 20 g) for neutralization. The effluents from the column, which were confirmed to be neutral using a pH test paper, totaled 500 mL with distilled water. The solution was diluted by a factor of 10 with distilled water for the HPLC analyses. The suspension of CFPN with or without MG in a centrifuge tube was completely transferred into a beaker using 80 mL of methanol. The suspension was sonicated, centrifuged, and neutralized by the same procedures as for AMX. The effluents from the column totaled 200 mL with methanol. The solution was diluted by a factor of 50 with methanol for the HPLC analyses. These treatments for the suspensions were rapidly (within 30 min) completed.

### HPLC

A Shimadzu HPLC system with a photodiode array detector (Kyoto, Japan) was used [24]. A reversed-phase Atlantis T3 column (250 × 4.6 mm i.d., 5 μm; Waters, Milford, USA) fitted with a guard column (20 × 4.6 mm i.d., 5 μm) was used. The composition of the mobile phases was determined, modifying those in the literature [25]. The composition of the mobile phase was 0.1% TFA/acetonitrile (84/16) for AMX and 0.1% TFA/acetonitrile (62/38) for CEPN, respectively. The wavelengths used to draw the chromatograms of AMX and CFPN were 230 and 262 nm, respectively. The analyses were performed at a flow rate of 1.0 mL/min at 25 °C. The final solutions (10 µL) prepared above were injected after being filtered using the membrane filter. The concentrations of the samples were measured using the absolute calibration method based on the peak areas. The amounts of medicinal ingredients in the simple suspensions were determined from the concentrations and the percentages of the measured values to the labeled amounts of the formulations (recovery rates) were calculated. An Atlantis T3 column (250 × 10 mm i.d., 5 μm; Waters, Milford, USA) equipped with a guard column (10 × 10 mm i.d., 5 μm) was used as a semi-preparative column for the isolation of the degradation products, where the composition of the mobile phase was the same as mentioned above and the flow rate was maintained at 4.8 mL/min.

### Validation method of HPLC analysis

Standard stock solutions of amoxicillin (616 µg/mL) and cefcapene pivoxil hydrochloride (656 µg/mL) were prepared in distilled water and methanol, respectively. The solutions for the validation were prepared from stock solutions. The calibration curves were constructed by triplicate analysis of samples with five concentrations whose ranges were wide enough for the quantitation of the medicinal ingredients. The calibration curves exhibited good linearity with the coefficients of determination (*r*^2^) being greater than 0.999 (Supplemental Table 1). Quality control samples were prepared at three concentrations (middle, high, low) within the ranges of the calibration curves. The precision was determined by the intra- and inter-day variations through repeated analyses (*N* = 5) of the quality control samples on three different days. The relative standard deviations of the measured values were less than 2.0% for the intra- and inter-day variations (Supplemental Table 2). Limit of detection (LOD) and limit of quantitation (LOQ) were determined based on the following equations, where S and S.D. are the slope and residual standard deviation of regression line at low concentrations near to LOD and LOQ, respectively. The LOD and LOQ of amoxicillin were 25.8 ng/mL and 78.3 ng/mL, respectively, and those of cefcapene pivoxil were 3.3 ng/mL and 10.0 ng/mL, respectively (Supplemental Table 1). All of the validation parameters were within the acceptance ranges.

$$\:LOD\hspace{0.17em}=\hspace{0.17em}3.3\:\times\:\:S.D.\:/\:S$$

$$\:LOQ\hspace{0.17em}=\hspace{0.17em}10\:\times\:\:S.D.\:/\:S$$

### Structural elucidation of degradation products from amoxicillin

The aqueous supernatant of the co-suspension of AMX and MG was concentrated using a rotary evaporator at room temperature. The residue was dissolved with distilled water (4 mL) and portions (300–500 µL) of the solution were injected into the HPLC apparatus with the semi-preparative column. The eluates corresponding to the peaks of the degradation products were collected and directly analyzed with a micro TOF-Q spectrometer (Bruker Daltonics, Kanagawa, Japan) in order to obtain the high-resolution electrospray ionization mass spectra (ESI-MS) and tandem mass spectra (MS/MS). The eluates were also evaporated to dryness using a rotary evaporator at room temperature. The residue was dissolved in DMSO-*d*_6_, and the solution was analyzed by nuclear magnetic resonance (NMR) spectroscopy with an AVANCE 500 NMR spectrometer (11.75 T, Bruker Japan, Kanagawa, Japan) using 5-mm sample tubes at 300 K.

### Statistical analysis

All data are expressed as the mean ± standard deviation of three experiments. All statistical analyses were performed using one-way ANOVA followed by Tukey’s test to determine significance. The threshold for assessing significance was *p* < 0.05.

## Results

### Preparation of simple suspensions

**Fig. 1:**
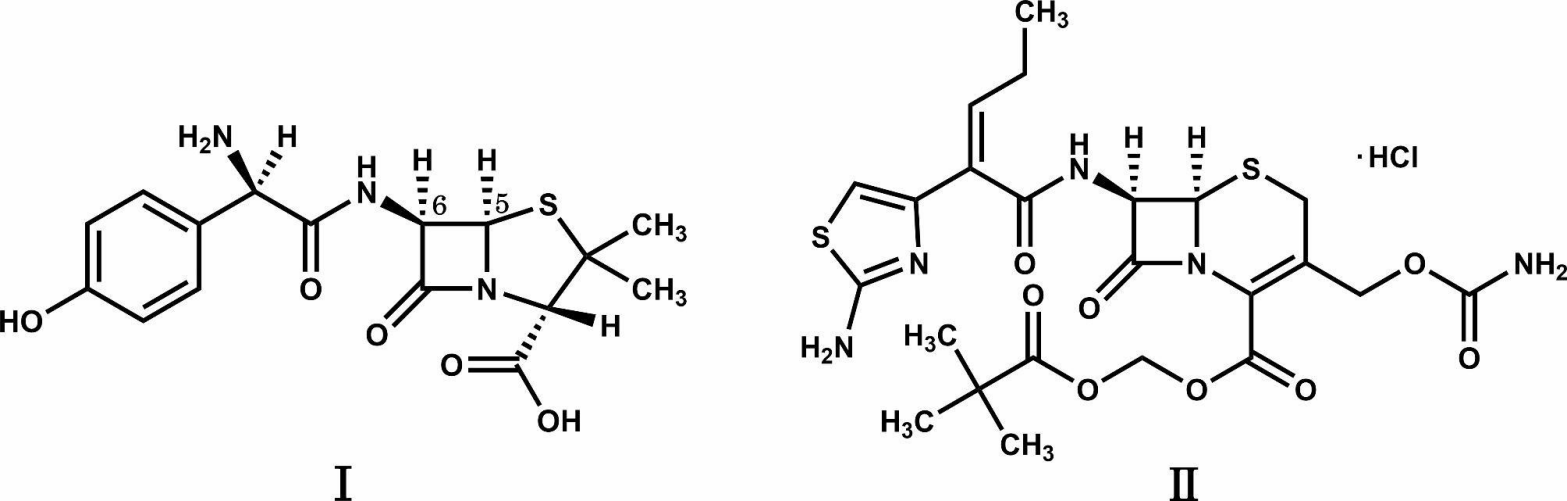
Chemical structures of amoxicillin (**I**) and cefcapene pivoxil hydrochloride (**II**)

The structures of the antibiotics examined are shown in Fig. 1. They have a β-lactam moiety labile to alkaline conditions and partial structures such as a carboxy group or an amino group, which are coordination groups. When the SSM was applied to AMX or CFPN, they were disintegrated within 10 min with or without MG, and their simple suspensions were successfully prepared. The pH values of the supernatants of the simple suspensions without MG were acidic, while those with MG were alkaline (Table 1).

**Table 1:** pH values of the supernatants of the simple suspensions prepared after soaking for 10 min^a^

Formulation	pH	
	without MG	with MG
AMX	5.6 ± 0.04	10.1 ± 0.03
CFPN	2.9 ± 0.03	9.7 ± 0.03

^a^Each formulation (one tablet or one capsule) was subjected three times to the SSM with or without MG (one tablet), and the pH values were measured. The values are shown as mean ± standard deviation

### HPLC analysis of suspensions

**Fig. 2:**
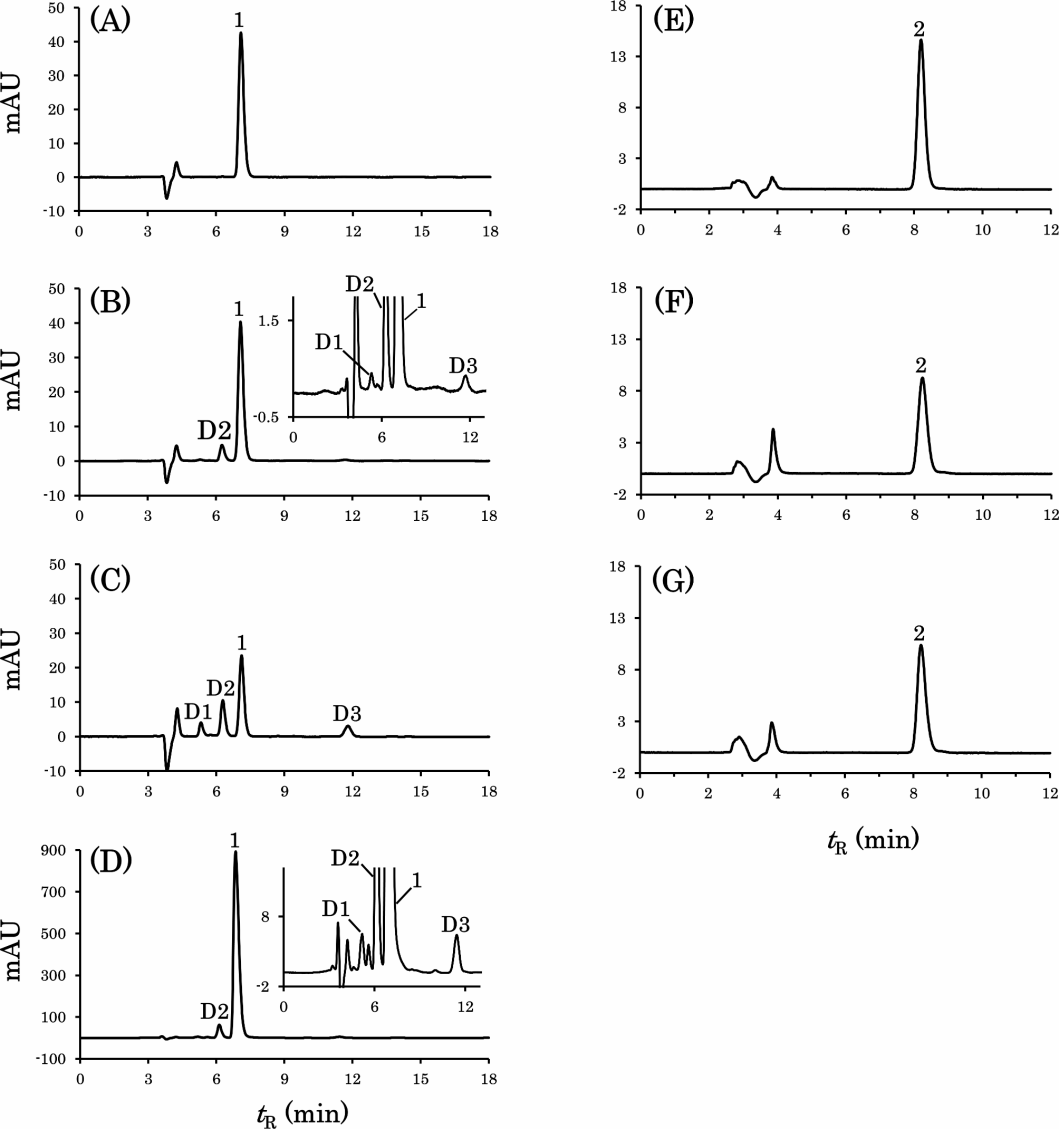
HPLC chromatograms of AMX (left) and CFPN (right) suspended with or without MG under the SSM conditions. The suspensions of the following formulations were prepared and treated and the resulting solutions were analyzed by HPLC. **A**: AMX; **B**, **C**, and **D**: AMX + MG; **E**: CFPN; **F** and **G**: CFPN + MG. **A**, **B**, **D**, **E**, and **F**: 10 min soaking; **C** and **G**: 5 h soaking. Panel **D** shows a direct chromatogram of the aqueous supernatant of the co-suspension with MG. The insets in panels **B** and **D** show the enlarged views. symbols: 1, amoxicillin; 2, cefcapene pivoxil; D1, D2, and D3, degradation product

The HPLC chromatograms obtained from the simple suspensions are shown in Fig. 2. Only the peak of amoxicillin (*t*_R_ 7.1 min) was observed for the suspension of AMX alone, whereas minor peaks of degradation products (*t*_R_ 5.3 min, 6.3 min, 11.8 min) were also observed after 10 min soakage for the co-suspension with MG (Fig. 2A and B). These degradation products are referred to as D1, D2, and D3, respectively. After 5 h soakage, the peak intensities of amoxicillin remarkably decreased and those of D1, D2, and D3 increased (Fig. 2C). The same degradation products were also observed when the aqueous supernatant of the co-suspension with MG was directly analyzed immediately after preparation of the suspensions (10 min soakage) (Fig. 2D). The peaks of D1 and D2 had shorter retention times than amoxicillin, suggesting that amoxicillin was transferred to more hydrophilic products due to hydrolysis of the β-lactam moiety. On the other hand, D3 was presumed to be a more hydrophobic product because of the longer retention time than amoxicillin. The absorption spectra of D1, D2, and D3 were considerably similar to those of amoxicillin. For CFPN, only the peak of cefcapene pivoxil (*t*_R_ 8.2 min) was observed when suspended with or without MG. Although no peak of the degradation product was observed, the peak intensity of cefcapene pivoxil decreased when co-suspended with MG (Fig. 2E-G).

The medicinal ingredients in the simple suspensions were quantified based on the HPLC peak areas and the recovery rates were determined (Table 2). The recovery rates of amoxicillin and cefcapene pivoxil from the suspensions without MG were approximately 99% after 10 min, and no significant decreases of the recovery rates were observed after 5 h. When AMX was suspended with MG, the recovery rate of amoxicillin significantly decreased after 10 min and decreased to approximately 50% after 5 h. When CFPN was suspended with MG, the recovery rates of cefcapene pivoxil were considerably lower than those of the suspensions without MG, and the reproducibility was relatively poor. The lower recovery rates of cefcapene pivoxil were considered to be due to the poor solubility of the molecular form in the alkaline water. Thus, in separate experiments, the co-suspensions of CFPN and MG were prepared in a flask, evaporated to dryness, and followed by redissolution with 100 mL of methanol. The HPLC analyses of the solution showed improved recovery rates of approximately 95%.

**Table 2:** Quantitative determination of the medicinal ingredients in the simple suspensions^a^

Formulation	without MG (%)		with MG (%)	
	after 10 min	after 5 h	after 10 mim	after 5 h
AMX	98.6±3.0	96.7±1.1	91.8±1.3^*^	50.6±1.0^#,†^
CFPN	98.9±1.8	96.7±1.2	74.8±4.6^*^	84.6±4.9^#,†^
			(95.1±3.8^b^)	(94.0±2.2^b^)

^a^The recovery rates are shown as mean ± standard deviation of three experiments

^b^The co-suspensions of CFPN and MG were evaporated to dryness and the residues were analyzed after being redissolved in methanol

^*^p < 0.05 vs. after 10 min soakage without MG

^#^*p* < 0.05 vs. after 5 h soakage without MG

^†^*p* < 0.05 vs. after 10 min soakage with MG

### Structural elucidation of degradation products from amoxicillin

**Fig. 3:**
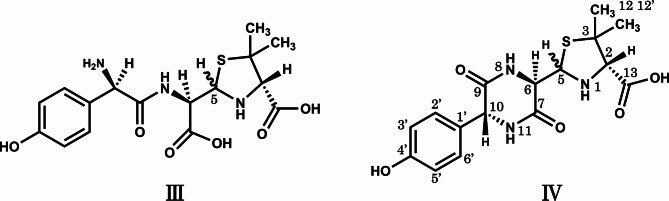
Chemical structures of the degradation products. Symbols: **III**, (5*R*)- and (5*S*)-amoxicilloic acid; **IV**, (5*R*)- and (5*S*)-amoxicillin diketopiperazine

The HPLC fraction containing the degradation product, D1, D2, or D3, was collected from aqueous supernatants of the co-suspensions of AMX and MG. Each fraction was confirmed to contain the desired degradation product by the HPLC analysis without concentration to dryness. However, when the fractions were evaporated to dryness and redissolved in acetonitrile/water (1/1), the fractions of D1 and D2 showed three HPLC peaks of D1, D2, and amoxicillin. These results indicated that D1 and D2 suffered the interconversion and the reversed change to amoxicillin during concentration under the acidic condition due to the TFA contained in the mobile phase. In contrast, the fraction of D3 still showed a single HPLC peak due to D3 even after evaporation. Thus, the D1 and D2 fractions were directly analyzed by mass spectrometer without concentration. The fraction of D1 showed ion peaks at *m*/*z* 384.1233 [M + H]^+^ and 406.1057 [M + Na]^+^, which indicated that the molecular formula of D1 was C_16_H_21_N_3_O_6_S. The fraction of D2 showed ion peaks at *m*/*z* 384.1221 [M + H]^+^ and 406.1042 [M + Na]^+^, which indicated that the molecular formula of D2 was the same as that of D1. The errors between the measured *m*/*z* and calculated one were less than 1.4 mDa. The molecular weight (383) of D1 and D2 was 18 (water molecule) greater than that of amoxicillin (C_16_H_19_N_3_O_5_S, 365), suggesting hydrolysis of the β-lactam moiety of amoxicillin. The MS/MS of D1 and D2 provided fragment ions at *m*/*z* of 367, 340, 323, 189, and 160 (Table 3), which was consistent with those of amoxicilloic acid [26] (Fig. 3). It is known that the configuration at the 5-position of amoxicillin is partially reversed after hydrolysis of the β-lactam ring, resulting in a mixture of diastereomers of amoxicilloic acid [27]. From these experimental results, D1 and D2 were shown to be amoxicilloic acid diastereomers.

**Table 3:** Accurate mass obtained from MS/MS of D1 and D2^a^

Formula	Calculated mass (m/z)	Experimental mass (m/z)	
		D1	D2
C_16_H_19_N_2_O_6_S	367.0958	367.0980 (-2.2)	367.0949 (0.9)
C_15_H_22_N_3_O_4_S	340.1326	340.1332 (-0.6)	340.1309 (1.7)
C_15_H_19_N_2_O_4_S	323.1060	323.1078 (-1.8)	323.1054 (0.6)
C_7_H_13_N_2_O_2_S	189.0692	189.0704 (-1.2)	189.0689 (0.3)
C_6_H_10_NO_2_S	160.0427	160.0433 (-0.6)	160.0426 (0.1)

^a^The applied collision energies for D1 and D2 were 16 eV and 18 eV, respectively. The errors (mDa) are shown in parentheses

**Fig. 4:**
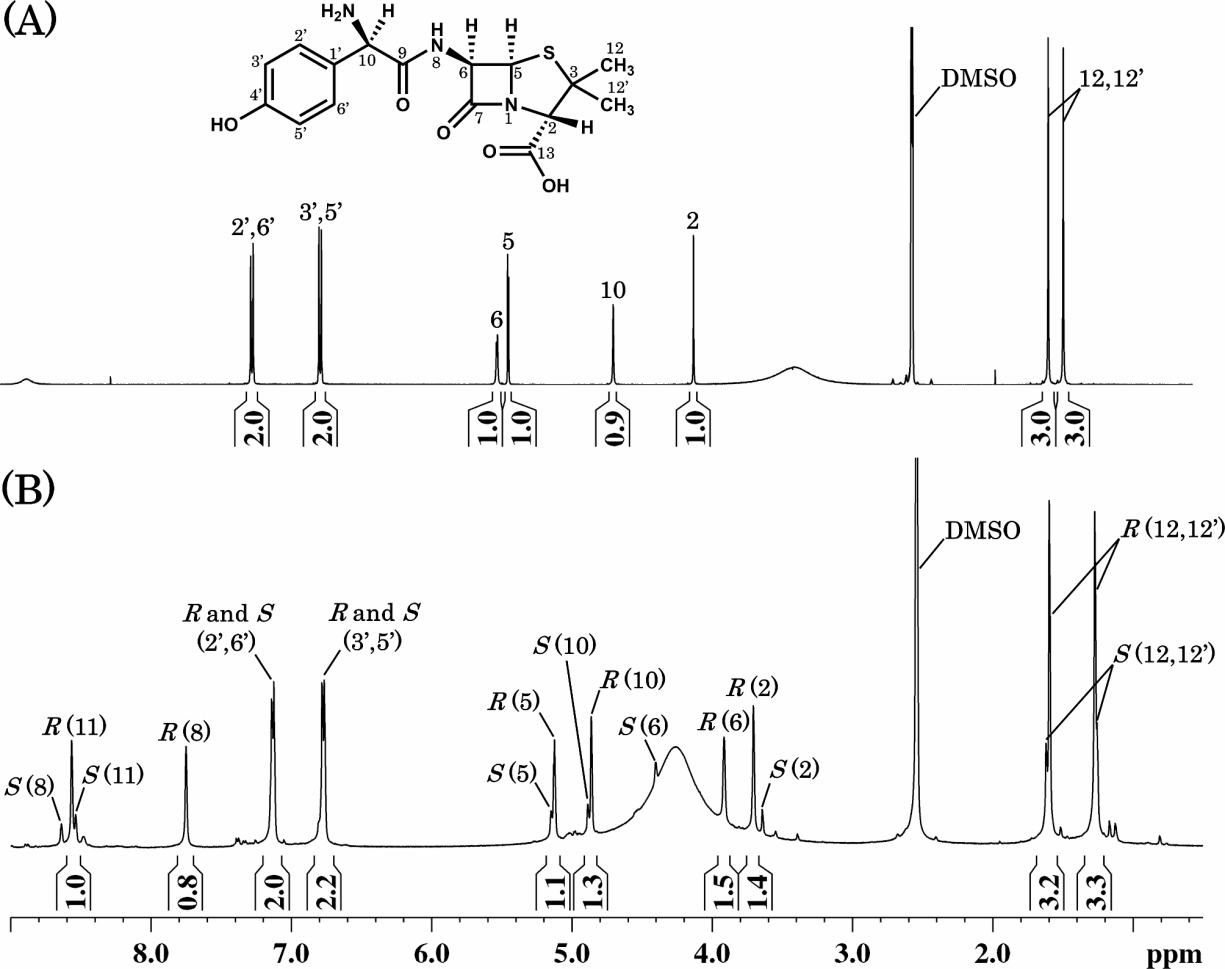
^1^H NMR spectra of amoxicillin (**A**) and its degradation product (D3) isolated from the co-suspension of AMX and MG (**B**). The spectra were measured in DMSO-*d*_6_. The major signals due to (5*R*)-amoxicillin diketopiperazine were observed with the minor ones due to the (5*S*)-diastereomer. The numerical characters on the spectrum (**B**) show assignments of the signals to the positions of the chemical structure of amoxicillin diketopiperazine (see Fig. 3). The integral values are shown under the ^1^H signals

When the ^1^H NMR spectrum of D3 was measured, signals due to the 5- and 6- positions of the β-lactam ring of amoxicillin were found to be shifted to a higher magnetic field (Fig. 4), suggesting hydrolysis of the β-lactam moiety. The accurate mass spectrum of D3 showed an ion peak at *m*/*z* 388.0916 [M + Na]^+^ which indicated that the molecular formula of D3 was C_16_H_19_N_3_O_5_S (calculated mass *m*/*z* 388.0938 with error of 2.2 mDa). It has been reported that amoxicillin in ammonia water (pH 10) degrades to the diastereomeric mixture of amoxicillin diketopiperazine (Fig. 3), which is the structural isomers of amoxicillin [28]. The ^1^H NMR spectrum of D3 was identical to the spectral data of amoxicillin diketopiperazine diastereomers in the literature [29, 30], and the individual signals were assigned as shown in Fig. 4. The structure was also supported by ^13^C NMR and two-dimensional NMR data (Supplemental Figs. 1, 2, and 3).

## Discussion

A variety of studies about the chemical stability of amoxicillin in alkaline aqueous media have been performed [31–33] since this drug is often used as a representative penam type β-lactam antibiotic and it is on the WHO model list of essential medicines [34]. In the forementioned reports, the chemical stability was found to vary depending on the media, temperature, time, and mixed substances. However, no information is available on the chemical stability of amoxicillin in alkalified simple suspensions. MgO is a representative alkaline drug which is widely used as a laxative and thus frequently ingested with other drugs. Thus, the investigation of the chemical stability of amoxicillin (in capsule form) in concomitant simple suspensions alkalified by MgO is of great significance for clinical practice. Cefcapene pivoxil is also a representative cephem type β-lactam antibiotic and frequently used. However, there have been no reports on the chemical stability of cefcapene pivoxil under alkaline aqueous conditions.

The present experimental results showed that amoxicillin was remarkably degraded by longer immersion even under the mild basicity of a co-suspension with MG. Although the soaking time to prepare simple suspensions is normally 10 min, in this study 5 h soaking time was also used, because simple suspensions are occasionally left for a long time due to business reasons [18, 35].

It is important to identify impurities and degradation products of drugs (formulations) in terms of efficacy and toxicity in pharmaceutical manufacturing settings [36]. Basically, the same seems to apply when drugs (formulations) are processed as in the SSM in clinical settings. The degradation products were found to be amoxicilloic acid and amoxicillin diketopiperazine, as in other alkaline conditions of the reports mentioned above. The degradation means the disappearance of the antibacterial action due to the β-lactam ring. Additionally, the degradation products may have some toxicity [37]. Thus, a co-suspension of AMX and MG should be avoided. Other penam type β-lactam antibiotics such as ampicillin, bacampicillin, and sultamicillin are presumed to also be degraded in the co-suspension with MG because they have the primary amino group in their side chains as in amoxicillin.

Simple suspensions need to be properly treated in order to correctly evaluate the stability of medicinal ingredients. In this study, the whole simple suspensions were treated with excess amounts of solvents to completely dissolve the medicinal ingredients. In addition, based on a previous report [17], the suspensions were neutralized with weakly acidic cation exchange resin to stop base decomposition. The use of methanol should be carefully considered for the analytical treatment of simple suspensions under alkaline conditions when a carboxy group is formed by the degradation of the medicinal ingredients [17]. When methanol was used for the treatment of the co-suspensions of AMX and MG, an HPLC peak of amoxicilloic acid methyl ester was observed in addition to those of amoxicillin and the degradation products (Supplemental Fig. 4). The methyl ester was considered to be formed by the methanolysis of amoxicillin, which resulted in overestimation of the degradation rate. Such a reaction of amoxicillin has also been reported in an alkaline aqueous methanolic solution [28]. Thus, in this study, the chemical stability of amoxicillin was investigated without using methanol for the analysis.

The suspensions of CFPN were treated with methanol since cefcapene pivoxil hydrochloride was easily soluble in the solvent. Although the recovery rate of cefcapene pivoxil was low in the co-suspensions with MG, no degradation peak was observed. The reason for the low recovery was found to be the low water solubility of the molecular form generated in the alkaline condition. These experimental results showed that cefcapene pivoxil was virtually stable in the co-suspensions with MG. To the best of our knowledge, there have been no reports on the chemical stability of cefcapene pivoxil under alkaline conditions. Moreover, to date, there is only one report about the stability of β-lactam antibiotics in the co-suspension with MG, where cefpodoxime proxetil was found to considerably degrade [16]. The results of the report are completely different from ours although the drug has the same cephem skeleton as cefcapene pivoxil and the two drugs are structurally very similar. The reported degradation of cefpodoxime proxetil may be due to the experimental conditions as well as the slight differences in structure.

This study dealt with the specific generic drugs of amoxicillin and cefcapene pivoxil hydrochloride. The pharmaceutical additives for amoxicillin differ depending on manufacturer and preparation. However, results similar to this study can be considered possible since the basicity of the co-suspensions is dependent on a large amount of MgO used. In addition, the additives for cefcapene pivoxil hydrochloride are common regardless of manufacturer.

## Conclusions

In this paper, the respective chemical stability of amoxicillin and cefcapene pivoxil hydrochloride in their preparations was investigated in an SSM with MgO. Amoxicillin was partially degraded, indicating that co-suspension with MgO should be avoided. This study has also indicated the degradation risk of prolonged soaking. No degradation product of cefcapene pivoxil hydrochloride was detected in the co-suspension with MgO. However, there are concerns about the passability of the feeding tube and the effect on absorption after administration because the generated molecular form has extremely low water solubility. The findings of this study should be informative for other generic drugs.

## Electronic supplementary material

Below is the link to the electronic supplementary material.

Supplementary Material 1

Supplementary Material 2

Supplementary Material 3

Supplementary Material 4

Supplementary Material 5

Supplementary Material 6

## Data Availability

Not applicable.

## Abbreviations

AMX: Amoxicillin capsules 250 mg
CFPN: Cefcapene pivoxil hydrochloride tablets 100 mg
DMSO-*d*_6_: Deuterated dimethyl sulfoxide
ESI-MS: Electrospray ionization mass spectra
HPLC: High-performance liquid chromatography
LOD: Limit of detection
LOQ: Limit of quantitation
MG: Magmitt tablets 330 mg
MS/MS: Tandem mass spectra
NMR: Nuclear magnetic resonance
SSM: Simple suspension method
TFA: Trifluoroacetic acid
