# Supplementary material for: Study on the chemical stability of β-lactam antibiotics in concomitant simple suspensions with magnesium oxide
Source: J Pharm Health Care Sci. 2024 Nov 18;10:73. doi: 10.1186/s40780-024-00396-0 (PMC11572518; doi:10.1186/s40780-024-00396-0)
Supplement: Supplementary file 1 — Supplementary Material 1 [file 40780_2024_396_MOESM1_ESM.docx]

Supplemental Table 1 Calibration curve, LOD, and LOQ

| Drug | Slope | Intercept | *r* ^2^ | Concentration range (μg/mL) | LOD (ng/mL) | LOQ  (ng/mL) |
| --- | --- | --- | --- | --- | --- | --- |
| amoxicillin | 13941 | −4083 | 0.9992 | 7.7-123 | 25.8 | 78.3 |
| cefcapene pivoxil | 18712 | 1603 | 0.9992 | 2.3-36.0 | 3.3 | 10.0 |

S.D. and S of amoxicillin were 72.75 and 9.29, respectively.

S.D. and S of cefcapene pivoxil were 22.62 and 22.51, respectively.
