# Supplementary material for: Study on the chemical stability of β-lactam antibiotics in concomitant simple suspensions with magnesium oxide
Source: J Pharm Health Care Sci. 2024 Nov 18;10:73. doi: 10.1186/s40780-024-00396-0 (PMC11572518; doi:10.1186/s40780-024-00396-0)
Supplement: Supplementary file 2 — Supplementary Material 2 [file 40780_2024_396_MOESM2_ESM.docx]

Supplemental Table 2 Intra- and inter-day variations^a^

| Drug | Concentration (μg/mL) |  | Intra-day | | | |  | Inter-day | | | |
| --- | --- | --- | --- | --- | --- | --- | --- | --- | --- | --- | --- |
|  |  |  | Mean (μg/mL) | Standard deviation | Relative standard deviation (%) | Bias^b^ (%) |  | Mean (μg/mL) | Standard deviation | Relative standard deviation (%) | Bias^b^ (%) |
| amoxicillin | 10 |  | 10.2 | 0.07 | 0.6 | 2.1 |  | 10.1 | 0.09 | 0.9 | 1.2 |
|  | 50 |  | 48.7 | 0.23 | 0.5 | −2.6 |  | 48.7 | 0.32 | 0.7 | −2.7 |
|  | 100 |  | 96.4 | 0.62 | 0.6 | −3.6 |  | 96.3 | 0.64 | 0.7 | −3.6 |
| cefcapene pivoxil | 2.3 |  | 2.3 | 0.01 | 1.0 | 3.0 |  | 2.3 | 0.03 | 1.2 | 2.4 |
|  | 18.0 |  | 18.4 | 0.07 | 0.4 | 2.3 |  | 18.5 | 0.15 | 0.8 | 3.3 |
|  | 35.9 |  | 37.7 | 0.11 | 0.3 | 4.8 |  | 37.0 | 0.76 | 2.0 | 3.0 |

^a^The quality control samples were analyzed five times using the HPLC system for the intra-day precision, and such experiments were performed on three different days for the inter-day precision.

^b^The differences between the sample concentrations and the measured ones.
