# Supplementary figures and images for: Study on the chemical stability of β-lactam antibiotics in concomitant simple suspensions with magnesium oxide
Source: J Pharm Health Care Sci. 2024 Nov 18;10:73. doi: 10.1186/s40780-024-00396-0 (PMC11572518; doi:10.1186/s40780-024-00396-0)

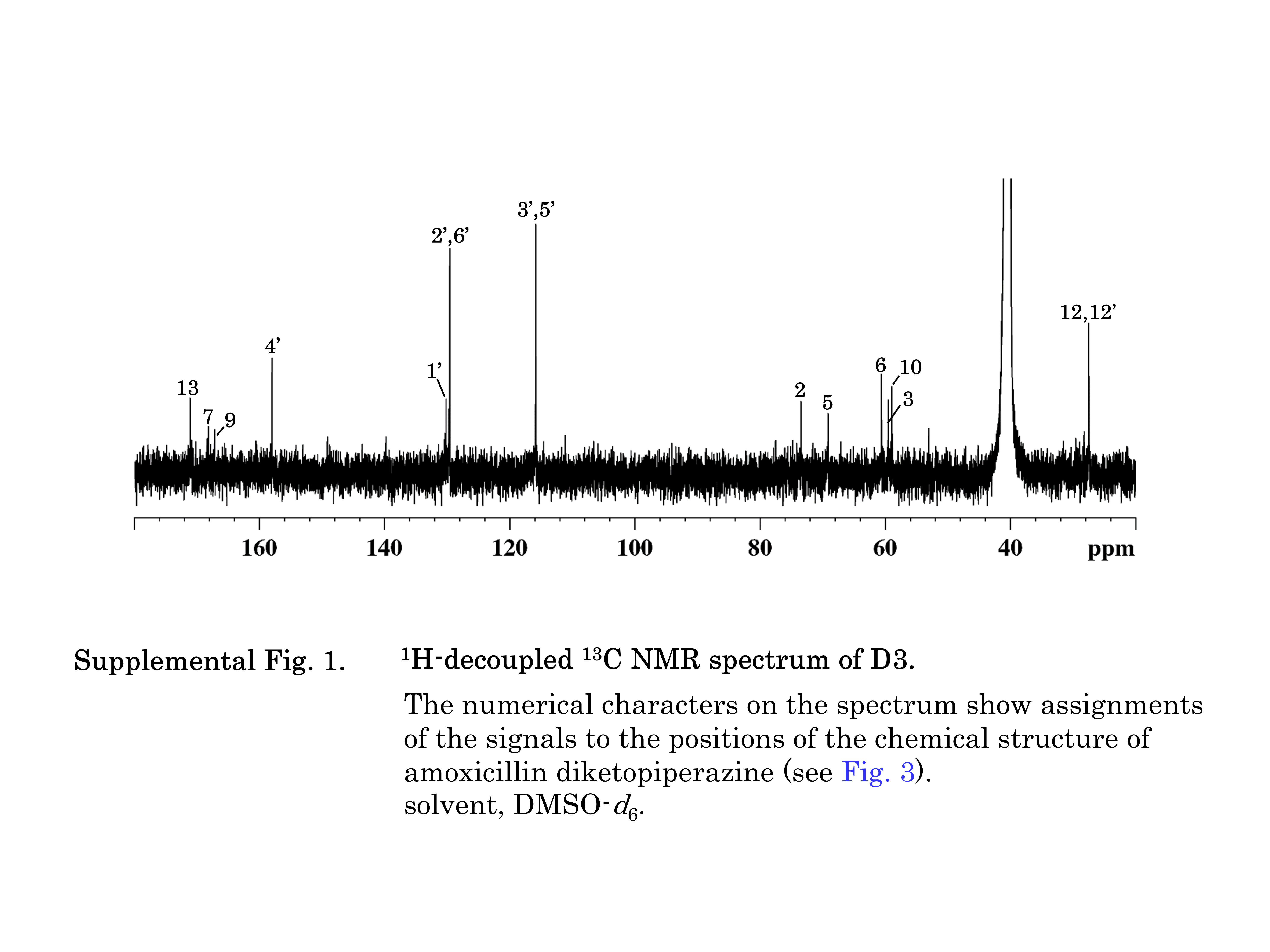

Supplement: Supplementary file 3 — Supplementary Material 3 [file 40780_2024_396_MOESM3_ESM.tif]

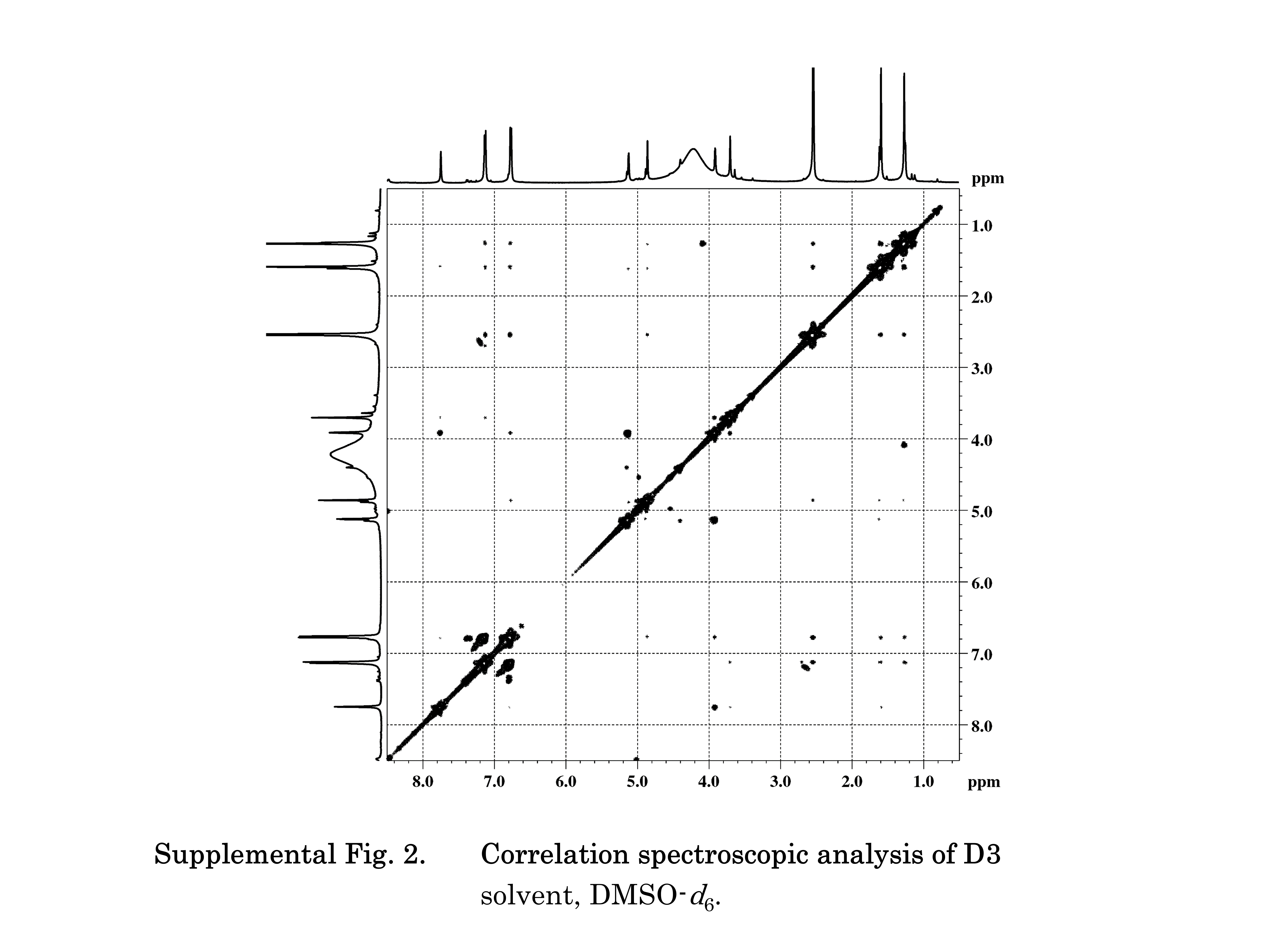

Supplement: Supplementary file 4 — Supplementary Material 4 [file 40780_2024_396_MOESM4_ESM.tif]

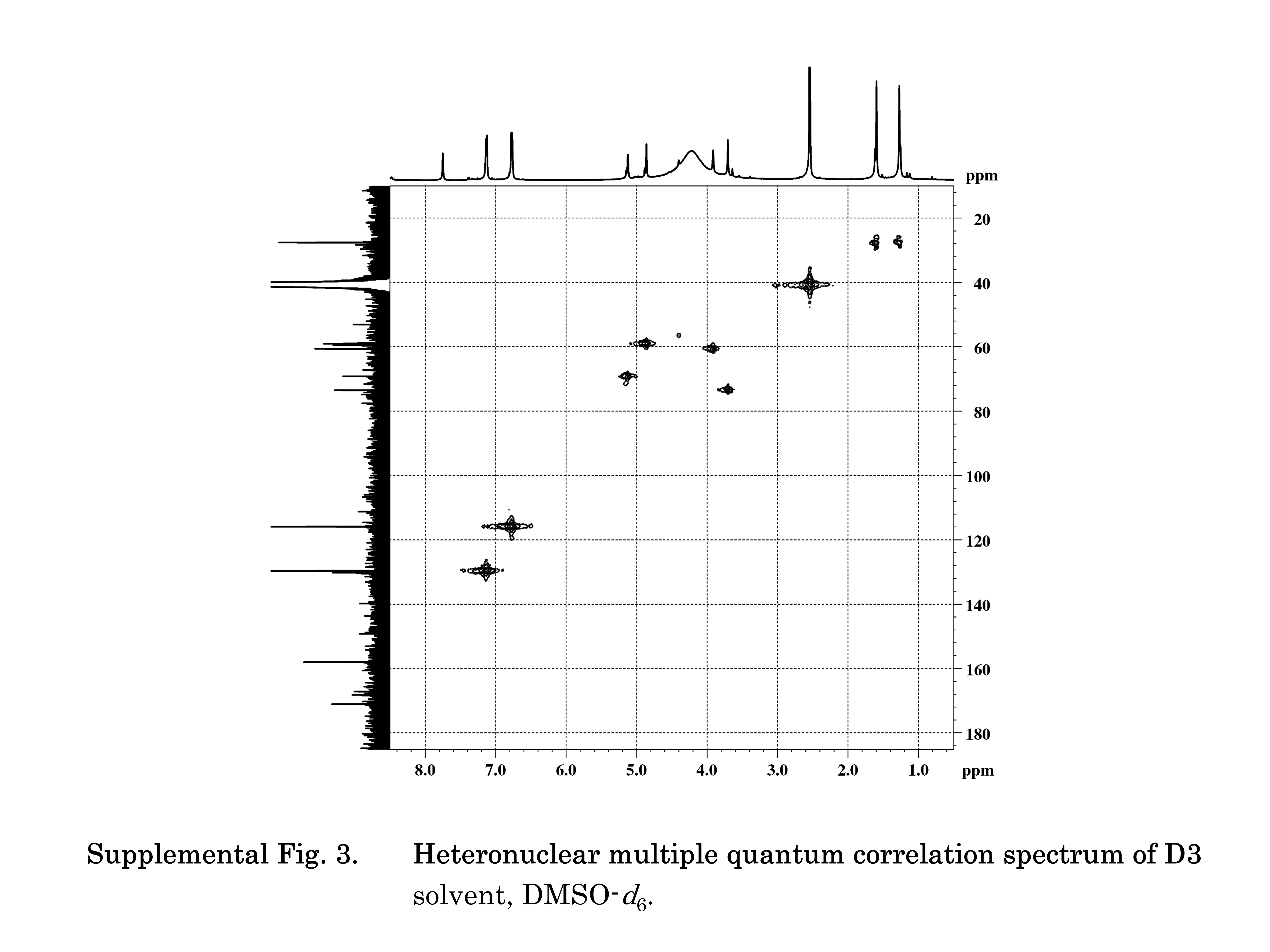

Supplement: Supplementary file 5 — Supplementary Material 5 [file 40780_2024_396_MOESM5_ESM.tif]

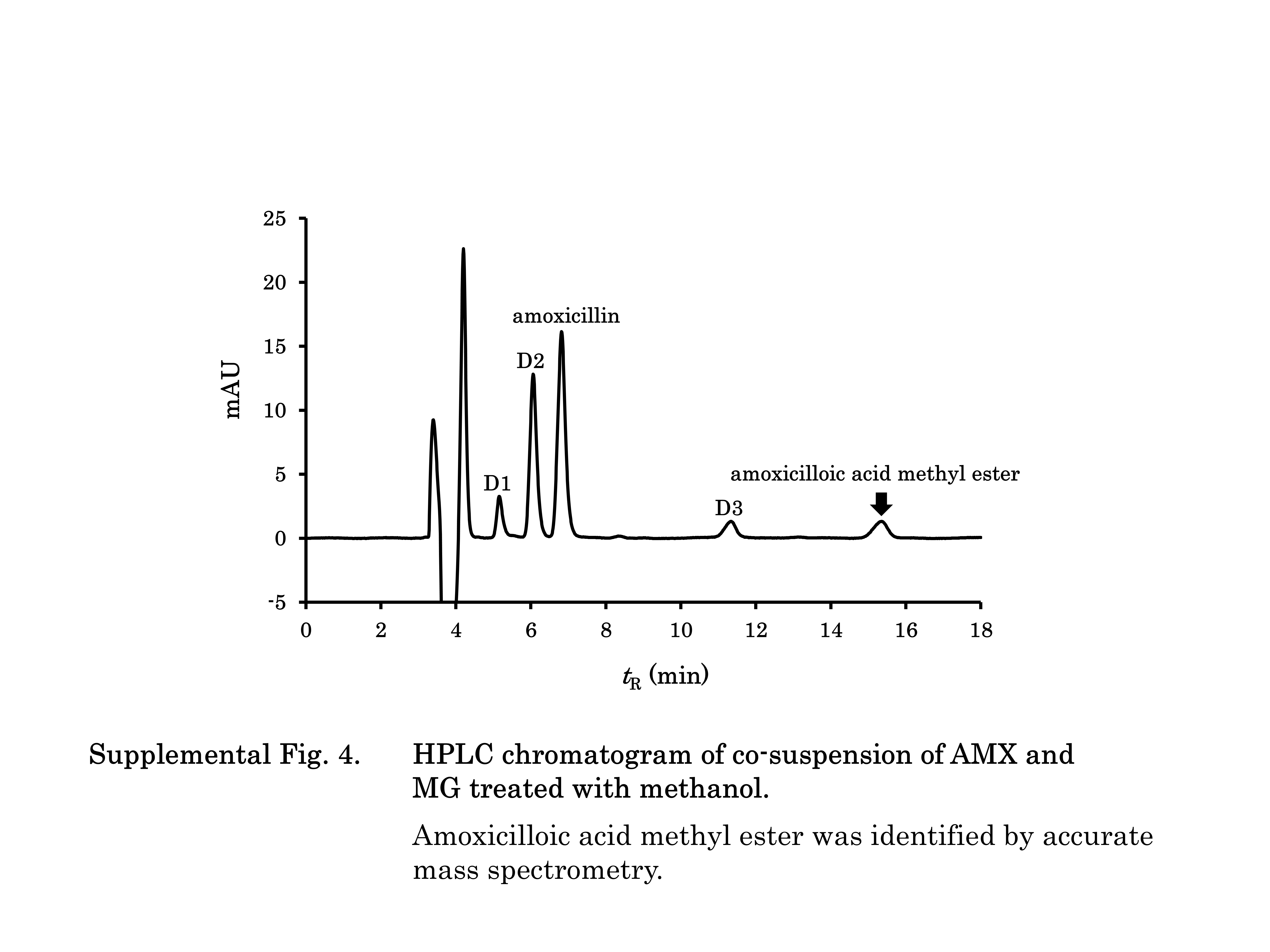

Supplement: Supplementary file 6 — Supplementary Material 6 [file 40780_2024_396_MOESM6_ESM.tif]
